# Online Haemodiafiltration Improves Inflammatory State in Dialysis Patients: A Longitudinal Study

**DOI:** 10.1371/journal.pone.0164969

**Published:** 2016-10-26

**Authors:** Ines Rama, Ines Llaudó, Pere Fontova, Gema Cerezo, Carlos Soto, Casimiro Javierre, Miguel Hueso, Nuria Montero, Alberto Martínez-Castelao, Juan Torras, Josep M. Grinyó, Josep M. Cruzado, Nuria Lloberas

**Affiliations:** 1 Nephrology Department, Bellvitge University Hospital, IDIBELL. Barcelona, Spain; 2 Consorci Sanitari del Garraf. Sant Antoni Abad Hospital, Vilanova i la Geltrú, Barcelona, Spain; 3 Department of Physiological Sciences II, School of Medicine, University of Barcelona, Barcelona, Spain; University of Kentucky, UNITED STATES

## Abstract

**Background:**

Patients undergoing conventional hemodialysis (C-HD) present a greater immuno-inflammatory state probably related to uremia, sympathetic nervous system (SNS) activation and /or membrane bioincompatibility, which could improve with a technique-switching to online hemodiafiltration (OL-HD). The antigen-independent pathway activation of this modified immunologic state turns dendritic cells (DC) into an accurate cell model to study these patients. The aim of this study is to further evaluate the immune-inflammatory state of patients in C-HD assessed by DC maturation.

**Methods:**

31 patients were submitted to C-HD and after 4 months switched to the OL-HD technique. Monocytes-derived DCs from HD patients were cultured in the presence of IL-4/GM-CSF. DC-maturation was evaluated by assessing the maturation phenotype by flow cytometry (FACs). DCs-functional capacity to elicit T-cell alloresponse was studied by mixed leucocyte reaction. Cytokine release was assessed by FACs and SNS was evaluated measuring renalase levels by ELISA.

**Results:**

An up-regulation of maturation markers was observed in C-HD DCs which induced two fold more T cells proliferation than OL-HD DCs. Also, C-HD-mDCs presented with over-production of pro-inflammatory cytokines (IL-6, IL-1β, IL-8, IL-10 and TNF-α) compared with OL-HD-mDC (P<0·05). Results were correlated with clinical data. When SNS was evaluated, hypotension events and blood pressure were significantly lower and renalase levels were significantly higher after conversion to OL-HD. Diabetes mellitus type 2 patients also found beneficial reduction of mDC when converted to OL-HD compared to non-diabetics.

**Conclusions:**

OL-HD could interfere with immuno-inflammatory state in HD patients with an improvement of renalase levels as potential key mediators in the mechanistic pathway of down-regulation of DC maturation.

## Introduction

Inclusion in the dialysis program involves an annual mortality of individuals between 13 and 20%, with an annual cardiovascular mortality of 9%. Part of the high risk of mortality and cardiovascular disease in dialysis patients has been attributed to baseline inflammatory status [1–4]. In addition, there is an increase in basal concentrations of pro and anti-systemic inflammatory cytokines in patients with renal replacement therapy [5] or by bioincompatibility with the dialyzer membrane, endotoxemia, infections and clinical events [6–10]. An activation of SNS has also been described [11, 12].

Accurate basic research studies and randomized controlled studies are needed to analyze the pathophysiology of the inflammation. Dendritic cells (DC) are potent antigen presenting cells, essential to trigger and to maintain immune response**s** (IR), able to link innate IR to adaptive IR. Our group previously demonstrated that DC can be activated by a non-antigen dependent stimulus, such as hypoxia [13–15], and thus, susceptible to be modulated by uremia and stress (SNS). Vitamin D also has an inhibitory effect on the adaptive immune system by regulating the function of DCs [16–18]. Thus, DC maturation could be a tool to clarify the triangle inflammation-SNS-uremia. In fact, chronic inflammation based on an increase of proinflammatory cytokines in patients on HD could trigger the function and activation of DCs [19].

Emerging evidence from other studies [20] suggests that the lack of removal of medium-sized molecules by C-HD may contribute to an increased inflammatory state supported by the data analysis of the CONTRAST study [21]. C-HD offers excellent clearance of small molecules, however, the morbidity and mortality in the dialysis population reaches inappropriately high percentages in relation to the general population. In fact, OL-HD combines both diffusion and convection, using high flux membranes and replacing the high convective volumes generated by ultrapure dialysis fluid, allowing the removal of medium size molecules by diffusion [22, 23]. This greater removal of uremic toxins could have an impact in reduction of inflammation and of SNS-activation, which could be related to DC modulation. In fact, it has been reported that patients treated with OL-HD showed lowered percentage of peripheral blood myeloid DC [24, 25].

This study confers an original approach by monitoring the inflammatory state of the patient**s** in OL-HD versus C-HD assessed by the functional study of DCs maturation and the antigen independent stimuli such as SNS which could provide better understanding of the potential benefits of OL-HD. For this purpose, in this study we evaluate for the first time the role of DCs in the inflammatory state which is inherent to patients with ESRD undergoing replacement therapies for the first time, comparing both techniques: C-HD and OL-HD and also assessing the impact of metabolic and SNS activation on DC modulation.

## Methods

### Patients

A total of 31 stable HD patients were included between May 2013 and May 2014. All patients had been treated in Bellvitge Hospital and the satellite dialysis center of Vilanova, for at least 6 months before inclusion and remained on the same technique of HD during the whole study. Twenty nine of the 31 patients finished the study. Two patients dropped out during the follow-up (one caused by influenza AH1N1 and one due to for pulmonary infection). Patients with active infection and neoplasic disease were excluded. Informed consent was obtained from all patients following the ethics committee of the hospital. Six patients undergoing kidney disease in pre-dialysis state were included as control. The follow-up was divided in two periods: Period 1 based on 4 month for high flux hemodialysis (C-HD) and Period 2 based on 4 months for online hemodiafiltration (OL-HD).

At inclusion, all patients had been on C-HD for at least 6 months and using polysulfone membranes (1.8 m2, HF80; Fresenius Medical Care, Bad Homburg, Germany). OL-HD was performed with ultrapure bicarbonate-buffered dialysate three times a week. Kt/V was determined at midweek of the last week. Analysis of the dialysis system always revealed absence of bacteria (<0.1 colony-forming units/ml) or bacteriological contaminant products (endotoxin levels <0.03 endotoxin units/ml) during the whole period of the study. The dialysis characteristics were similar in the two modalities, except for a higher convective transport in the OL-HD than in C-HD. In OL-HD, the mean volume infused was recorded and was found to be >20 L per session. The composition of dialysate was the same in both OL-HD as in C-HD and also in the of reinfusate in OL-HD (sodium 138–140mmol/L, potassium 1.5–2.0 mmol/L, calcium 1.25–1.75 mmol/L, magnesium 0.5 mmol/L, chloride 106–109 mmol/L, bicarbonate 34–37 mmol/L, acetate 3–4 mmol/L, and glucose 1.0 g/L).

Patients underwent C-HD with dialyzer surface area of 1.7 to 2.1 m^2^ membranes and surface area was maintained when patients were converted to OL-HD. Dialysis efficiency was estimated using eKt/V ratio. All patients had a native arteriovenous fistula and two patients showed an arteriovenous graft. Basal data were collected to record age, gender, weight, height, underlying renal disease, dialysis vintage, history of transplantation, diabetes, comorbid conditions, current medication, blood pressure and hypotension. Hypotensive events were defined as a decrease in systolic blood pressure of ≥20 mm Hg or a decrease in MAP of 10 mm Hg associated with symptoms. None of the patients were on anti-inflammatory or immunosuppressive drugs during the 3 months before inclusion and for the whole period of the study. Nine patients were diabetic. The therapeutic regimen, which consisted of erythropoietin (EPO), calcium-containing phosphate binders, antihypertensive drugs, and intravenous iron, was changed according to the clinical needs. Patients received EPO from the beginning of the study. EPO dosage was adjusted to maintain the hemoglobin target of 11.5 to 12 g/dl. All routine laboratory measurements were done using certificate assay methods. All patients received paricalcitol iv therapy during the study follow up, attending to medical needs.

Blood samples were obtained from all patients just before the first (long interval) dialysis session of the following period to study the effect after 4 months of OL-HD.

### Ethics statement

Human cells were obtained in accordance with protocols approved by the Ethics Committee of the Hospital Bellvitge of Barcelona (Barcelona, Spain) and in accordance with the principles of the Declaration of Helsinki. Written informed consent was obtained from the participants.

### Isolation and culture of cells

Whole peripheral blood samples obtained from HD patients and healthy subjects were collected in tubes that contained K3EDTA (9mL) immediately before dialysis treatment and processed within 1h of collection. Peripheral blood mononuclear cells (PBMCs) were isolated by density gradient centrifugation (Ficoll-PaqueTM PLUS; GE Healthcare, Uppsala, Sweden). 10x10^6^ cells were incubated for 2h at 37°C in 5% CO_2_ in 25cm^2^ flask plates. After washing, the adherent monocytes were cultured in complete medium that contained X-VIVOTM supplemented with 2% Human Serum (Sigma-Aldrich; Madrid, Spain) in the presence of IL-4 (500U/ml; Sigma) and granulocyte monocytes colony-stimulating factor (GM-CSF 1000U/mL; R&D Systems, Minneapolis, MN, US) for a total of 6 days at 37°C in a humidified atmosphere of 5% CO_2_, obtaining 90% DC purity at day 6. Analysis of different cellular subpopulations during differentiation and maturation of dendritic cells (DCs) and the gating strategy for DC population was described and validated in our previous studies [15].

### Dendritic cell phenotype analysis by flow cytometry

After 6 days of culture, different cell surface markers were analyzed by flow cytometry to study the maturation process of DC on C-HD and OL-HD patients. This cytometric analysis was made by FACS CANTO^TM^II and the data was analyzed using FACS DIVA software (Becton-Dickinson, San Diego, CA, US). In order to examine the DC maturation, the markers used were: CD80 (Clone BB1), CD83 (Clone HB15e) and HLA-DR (Clone G46-6) conjugated with fluorescein isothiocyanate (FITC) and CD40 (Clone 5C3), CD86 (Clone 2331 FUN-1), and CD54 (Clone HA58) conjugated with allophycocyanin (APC). The cytometric protocol and the combination of the antibodies used in our experiments were described by our group in previous studies [15, 26]. To assess the differentiation of monocytes along the culture, CD14 (Clone M5E2) FITC antibody was used. All the antibodies were mouse monoclonal from Becton-Dickinson Pharmingen.

### Allogeneic mixed leucocyte reaction. T-Cell proliferation

We then next tested weather DCs have an impact on T-cell proliferation. For this purpose, we performed a CFSE assay in order to analyze the effector function of DCs. We isolated DCs from C-HD/OL-HD and mixed them with frozen PBMCs from healthy volunteers by performing MLR [27, 28]. The allogenic PBMCs were labelled with CFSE (carboxyfluorescein diacetate succinimidyl ester; Molecular Probes, Madrid, Spain) and exposed to mature DCs from HD patients. Allogeneic CFSE-labelled PBMCs (2×10^5^) were co-cultured in 96-well round-bottom plates in the presence of DCs (2×10^4^) collected at the end of the 6-day culture at a 1:10 DCs-to-T-cell ratio. The mixt culture was incubated in RPMI medium (GE Healthcare Life Sciences HyClone Laboratories, Logan, UT, US) supplemented with 10% FBS (Cultek, Madrid, Spain), 100 U ml-^1^ of penicillin, 100 μgml^-1^ streptomycin and 2mM L-Glutamine at 37° and 5% CO_2_ conditions. After 5 days of culture, the cells were labelled with CD3 (Clone HIT3a) APC (Becton-Dickinson Pharmingen) to analyze T-cell proliferation by flow cytometry by FACS CANTO^TM^II and analyzed by FACS DIVA software.

### Quantification of cytokine production by DCs using cytometric bead array

After MLR, serum and supernatants from HD patients after MLR were collected. Cytokine levels of Interleukin-1β, -6, -8, -10, -12p70 and TNF-α were measured quantitatively by the cytometric bead array technique (CBA Human Inflammatory Cytokines Kit, Becton Dickinson). The levels were measured according to the manufacturer’s instructions. Flow cytometry analysis was performed using FACS CANTO and FACS DIVA software.

### Quantification of Renalase

SNS was analyzed by quantification of renalase. The enzyme-linked immunosorbent assay (ELISA) Kit (Uscn Life Science Inc. China) using a monoclonal antibody specific to renalase, was performed in cells supernatant and serum of HD patients (C-HD and OL-HD) in order to estimate the renalase concentration. The enabled protocol from manufacturer’s instructions was used.

### Statistical Analyses

Clinical and biological parameters were described using mean and standard deviation for continuous variables and frequencies for categorical variables. Differences between study group means were tested by paired t-test. A subgroup analysis was conducted on diabetic mellitus patients. Differences between diabetic and non-diabetic patients were tested by t-test.

Spearman correlation between renalase and number of hypotension events was performed. A linear regression model was estimated to test the association between renalase and systolic blood pressure and between convective volume in HD-OL and DC maturation. Also Pearson correlation was performed between renalase values and systolic blood pressure. P<0.05 was considered statistically significant. All statistical analyses were conducted using SPSS v17.

### Limitations of the study

This is a longitudinal study design with the methodological limitations of this approach. Thus, the possibility that the effect could be related to longer dialysis treatment period cannot be ruled out.

## Results

### 1. Analysis of DC populations under C-HD and OL-HD patients

At day 0, the analysis of different cellular subpopulations during the differentiation and maturation of DCs showed a high percentage of monocytes (CD14+) and the presence of several lymphocyte subtypes. During differentiation, the CD14+ population expressed DCs markers such as (HLA-DR+ and CD11c+) and the lymphocyte percentage diminished after removing the medium and replacing it with fresh culture medium. DC population was gathered in two subpopulations, depending on the degree of maturation according to the forward-/side-scatter profile and specific phenotypic markers established in our previous study [13, 15]. Cells changed their morphology, acquiring a stellate form, characteristic of the mature DC (mDC) shifting to the upper window depending on the dialysis technique.

**Fig 1:**
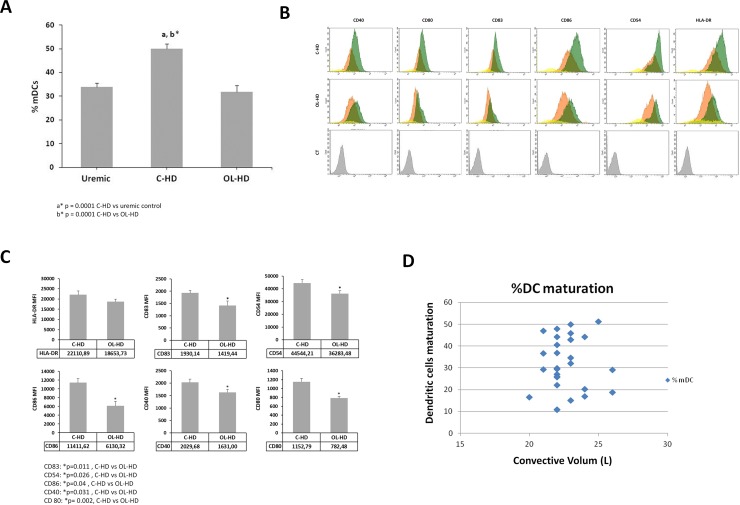
Dendritic cell maturation. **A.** Modulation of dendritic cell maturation in HD and uremic patients. DCs were obtained from HD and uremic patients and were cultured for 6 days. Expression of cell surface markers was evaluated by flow cytometry and the results were expressed as the percentage of matured DC. **B.** In the histogram, monocytes are represented in yellow, the immature DC in orange, the matured DC in green and the negative control in grey. MFI and expression of each marker are presented as mean ± SD. **C.** or Expression of cell surface markers evaluation by the mean fluorescence intensity (MFI) of different maturation markers as CD40, CD80, CD83, CD86, CD54 and HLA-DR in C-HD and OL-HD groups. **D.** Correlation between DCs maturation and the different convective volumes (D).

C-HD patients at baseline express higher percentage of mDCs compared to uremic patients pre-dialysis (50.06 ± 4.41 versus 28 ± 5.1; *p* = 0.001) S1 Table. After 4 months under OL-HD, these same patients showed a marked decrease in the percentage of mature DCs compared with C-HD status (31.91 ± 3.15; vs 50.06 ± 4.41; *p* = 0.001) (Fig 1A and S1 Table). Also, C-HD induced higher DC maturation, this was statistically significant compared to OL-HD analyzed as intensity of different maturation markers (CD40, CD80, CD83, CD86 and CD54) (Fig 1B and 1C). C-HD patients at baseline expressed a high percentage of mDCs compared to uremic patients pre-dialysis (50.06 ± 4.41 versus 28 ± 5.1; *p* = 0.001). After 4 months under OL-HD, these same patients showed a marked decrease in the percentage of mature DCs compared with C-HD status (31.91 ± 3.15; vs 50.06 ± 4.41; *p* = 0.001) (Fig 1A and S1 Table)., In addition, C-HD induced higher DC maturation, which was statistically significant compared to OL-HD analyzed as intensity of different maturation markers (CD40, CD80, CD83, CD86 and CD54) (Fig 1B and 1C). Maturation-DC degree was also analyzed in association with different convective volumes under OL-HD, no correlation was found between DCs maturation and the different convective volumes (Fig 1D).

### 2. T- cell proliferation in C-HD and OL-HD patients

**Fig 2:**
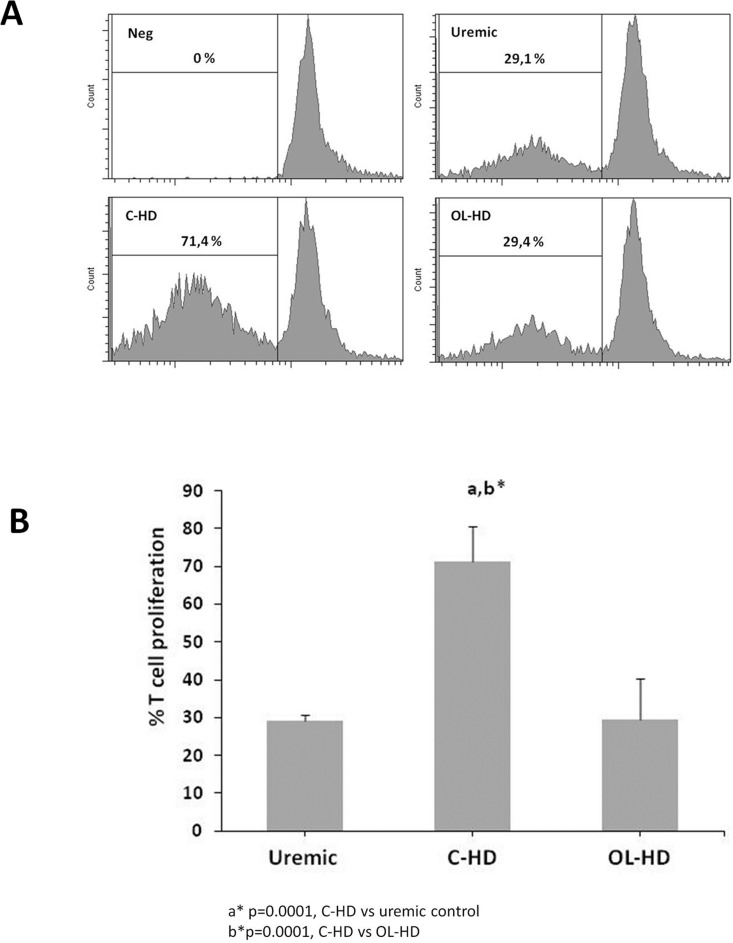
OL-HD reduces T-cell proliferation induction by DC in the MLR. **A.** Lymphocytes were stained with CFSE and exposed to mature DCs (Uremic control vs C-HD vs OL-HD) during 6 days of culture. **B.** Cell proliferation was determined by flow cytometry after labelling with CD3 antibody.

To address the functional impact of C-HD vs OL-HD on DCs, we then assessed the effects of DCs on lymphocyte proliferation by MLR. Matured DCs from C-HD (C-HD-mDC) significantly induced T-cell proliferation compared to matured DCs from OL-HD patients (2A). (OL-HD-mDC) (71.44±7.7 vs 29.46±7.75; *p =* 0.03). OL-HD matured DCs achieved similar T cell proliferation and uremic control (29.10±9.29) (Fig 2B and S1 Table).

### 3. Cytokine release in serum and in supernatant of PBMCs after MLR

**Fig 3:**
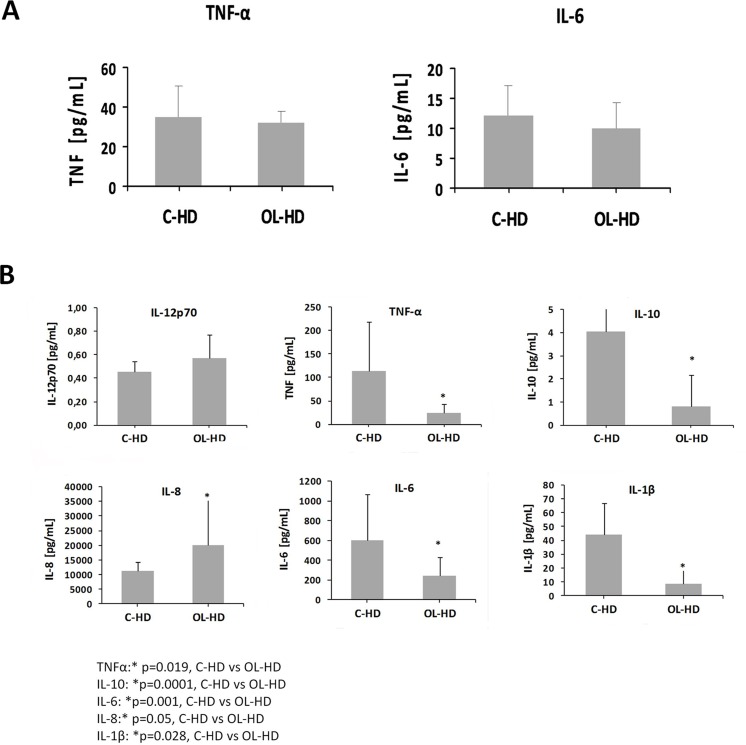
Cytokine release. **A.** Different pattern of cytokine release was obtained in OL-HD compared to C-HD. IL-6 and TNFα levels from serum. **B.** 1β, IL-6, IL-8, IL-10, IL-12p70 and TNFα secretion protein levels from cell supernatant after MLR were measured by cytometric bead array. The results are expressed in pg/ml ± SD.

Cytokine release of IL-6 and TNF-α from patients on C-HD did not present statistically significant differences compared to those on OL-HD (Fig 3A and S1 Table). On the other hand, cytokine release in the mixed culture with mDCs and lymphocytes showed a different pattern depending on the HD technique. PBMCs, mostly lymphocytes, stimulated by C-HD-mDCs presented statistically significant over-production of IL-1β, IL-6, IL-10 and TNF-α, related mainly to a T helper type 1 (Th1) response, compared with control (Fig 3B and S1 Table). In contrast, IL-8 was over-expressed in PBMCs exposed to OL-HD-mDCs, suggesting a switch to a Th2 response (Fig 3B and S1 Table).

### 4. Impact of OL/HD in diabetic and non diabetic patients

Diabetic C-HD patients showed higher percentage of dendritic cell maturation compared to non-diabetic patients C-HD (66.85±3.92 vs 48.13±4.62 respectively; *p* = 0.03). Interestingly, diabetic OL-HD patients showed a tendency towards lower dendritic cell maturation values but the switch C-HD to OL-HD reduced the difference in DC maturation between diabetics and non-diabetics respectively (44.16±7.54 vs 31.31±4.17; *p* = 0.164).

Diabetic patients under C-HD showed higher percentage of lymphocyte proliferation on CFSE after MLR, compared to diabetic patients under OL-HD (78.85±1.2 vs 12.7±2.6 respectively; *p* = 0.04); the switch of C-HD to OL-HD in non diabetic patients showed a slight reduction in lymphocyte proliferation by CFSE, not achieving statistical differences.

### 5. Biochemical and clinical parameters

Biochemical data were assessed by conventional venoclysis for parameters related to ESRD under replacement therapy before HD sessions. All relevant biochemical and clinical data are summarized in Tables 1 and 2; and S1 Table. Interestingly, there was a better management of systolic and diastolic blood pressure and less episodes of severe hypotension in OL-HD patients compared with C-HD (systolic TA. 130.33±3.5 vs 148.12±5.1; *p*<0.001; diastolic TA. 65.70±2.8 vs 72.87±1.9; *p* = 0.04) and severe hypotension episodes (0.12±0.6 vs 1.62±0.22; *p* = 0.001).

**Table 1:** Baseline characteristics.

Parameter	
Age	72±15
Gender (M/F)	17/14
Native arteriovenous fistula	30
Arteriovenous graft	1
ESRD ethiololgy	
• Diabetes	10
• Polycystic disease	3
• Autoimmune disease	0
• Nephrotoxicity	1
• Urologic	4
• Vascular	5
• Not known	8
Dialysis vintage (months)	46.54
Kidney transplant	1
Exitus	2
Number of patients	31

Table 1 shows demographic parameters of patients throughout the study. Twenty-nine of the 31 patients finished the study.

**Table 2:** Biochemical and clinical characteristics.

Parameter (Mean ± SD)	C-HD	OL-HD	P
Weight (Kg)	70.1 ± 13.6	70.4 ± 16.4	0.9392
Ferritina (μg/L)	615.35±87.04	586.53±96.83	0.15
% DC maturation	50.06 ±4.41	31.91 ± 3.15	0.001
CFSE	71.44±7.70	29.46±7.75	0.03
iPTH (Pmol/L)	28.76±33.65	22.00±18.16	0.25
Calcidiol (nmol/L)	34.54±2.01	46.65±10.20	0.20
Fosfate (mmol/L)	1.26±0.45	1.30±0.79	0.79
Albumin (g/L)	38.7±2.5	39.08±3.3	0.397
nPCR	1.08±0.09	1.10±0.09	0.201
PCR	15.057±16.18	12.802±15.8	0.48
Blood Pressure			
• Systolic	148.12±5.1	130.33±3.5	0.001
• Diastolic	72.87±1.9	65.70±2.8	0.04
• Hypotension events	1.62±0.22	0.12±0.6	0.001
Renalase (ng/ml)	103.12±6.43	162,83±10.39	0.0001
Hemoglobin (g/L)	11.58±2.43	12.67±1.65	0.37
Leukocytes (x10^9^ cells/L)	7158.00±370.45	6986.8±345.51	0.019
Lymphocytes (x10^9^ cells/L)	1364.61±97.11	1335.15±103.89	0.5
eKt/V ratio	1.58±0.33	1.62±0.35	0.12

Table 2 shows demographic and biochemical parameters of patients throughout the study. The follow-up was divided in two periods: Period 1 based on 4 months of high flux hemodialysis (C-HD) and Period 2 based on 4 months of online hemodiafiltration (OL-HD).

### 6. OL-HD benefit in renalase release

**Fig 4:**
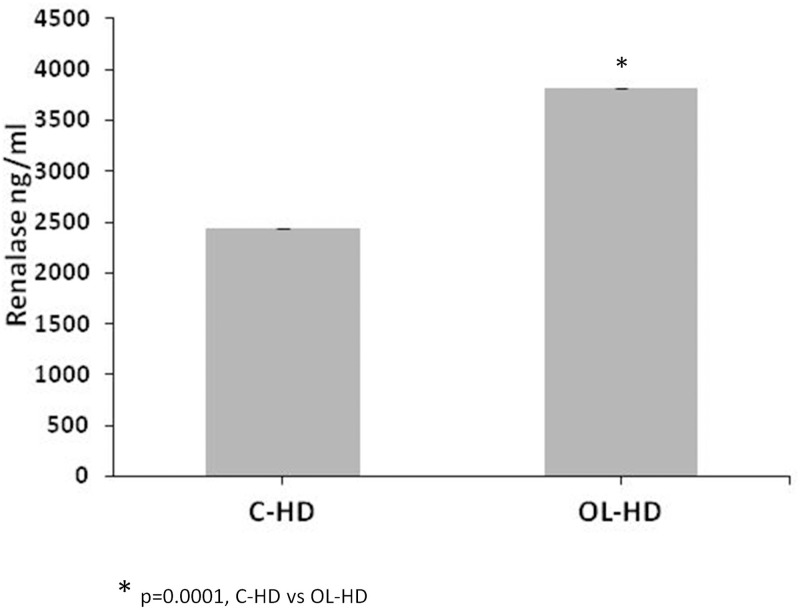
Renalase release is higher in patients following OL-HD than in those following CD-HD. C-HD and OL-HD patients’ serum renalase levels were measured by ELISA. The result**s** are expressed in ng/ml ± SD.

**Fig 5:**
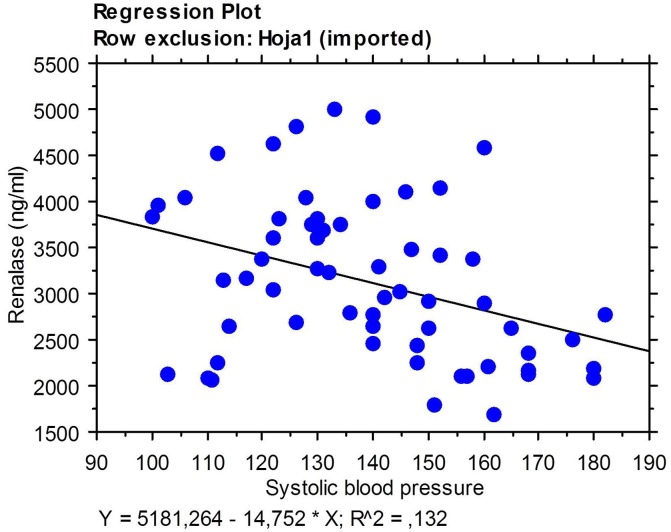
Correlation of serum renalase levels with hypotension events and systolic blood pressure control.

Renalase in C-HD patients serum levels were assessed by ELISA in serum of C-HD patients and in the same patients after four months of conversion to OL-HD. Interestingly; renalase levels were significantly higher for patients undergoing OL-HD (Fig 4 and S1 Table). In fact, there was a correlation of renalase levels with clinical parameters related to SNS. Higher renalase values correlated with lower severe hypotension events (-0,410 (Rho value) p = 0.0033) and better systolic blood pressure control (0,327 (Rho value) p = 0.0587). Indeed, renalase levels would be 160 fold higher for every reduction in systolic blood pressure of 10mmHg (-16,08 (beta value) (Fig 5).

## Discussion

Inflammation in patients with ESRD undergoing HD is an increasing concern for physicians and has been related to increase the rates of morbidity and mortality [29–31]. Interestingly, patients with ESRD in C-HD have frequent infections and a suboptimal response to vaccines; this is probably related to an immune inflammatory disorder associated either with uremia and / or nutritional status [32]. In this sense, OL-HD has been shown to improve cardio-protection and the immunologic system and reduces infection and mortality compared with C-HD [33]. A recent study showed that OL-HD compared with C-HD reduced the risk of mortality in ESRD patients [34].

As our group previously revealed, DCs are the most potent APCs in the initiation of an immune-inflammatory response, either after antigen or non-antigen-dependent stimuli as our group previously revealed [15]. An aberrant DC function in HD patients has also been recently suggested as a cause of impaired immune response [19] due to a strong alteration in DCs generation and maturation in ESRD [35]. Considering the capacity and the central role of DC, the present study extends the characterization of monocytes to DCs to evaluate for the first time the role of DCS in inflammation on C-HD. For this purpose, we evaluated the change in the morphology and phenotype of DCs in C-HD patients. As we have previously described, mDCs changed their morphology acquiring a characteristic stellate form based on a non-antigen stimulation of DCs as we have previously described. Thus, we made an attempt to evaluate whether uremic milieu and hemodialysis technique per se could trigger adaptative immune response through modulation of DCs. For this, maturation of monocyte-derived DCs from patients undergoing C-HD was evaluated and compared to monocyte-derived DCs of the same patients after four months under OL-HD therapy. Interestingly, percentage of mDC was lower in OL-HD patients, which decodes in a lower inflammatory state under this technique. According to our results, several studies have shown the positive effect of OL-HD on cardiovascular survival and co-morbidity of patients [33, 34, 36–39].

To evaluate DC function, an allo-mixed lymphocyte reaction was performed to analyze the capacity of DCs to stimulate proliferation of naive T cells. Functional impact of mDC showed a higher stimulatory capacity in allogeneic T-cell proliferation for patients exposed to C-HD than those under OL-HD. Patients with OL-HD achieved the same T cell proliferation than control-uremic patients although these reached higher values compared to healthy volunteers. In fact, it has been reported that aberrant mDCs in C-HD patients have have a higher T cell proliferation stimulatory capacity than that in control healthy volunteers [19]. Choi et al, [19] postulated that there is a possibility that chronic inflammation might affect DC function, resulting in abnormal allostimulatory capacity. Lymphocytes stimulated by mature DCs release a broad array of cytokines exerting a wide influence on the immune system. Depending on the HD technique, our results showed a different pattern in the cytokine release by lymphocytes after the mixed culture with mDCs. Therefore, lymphocytes stimulated by mDCs from C-HD patients showed a higher secretion of cytokines related to Th1 response compared with those stimulated by mDCs from OL-HD patients who mainly expressed a cytokine Th2 profile. These results support other studies which reveal that dysregulated cytokine network -anti-inflammatory (IL-10) and proinflammatory (IL-6 and TNF-α) cytokines- play an important role in the wasting syndrome of uremic milieu [35, 40].

Our novel results show that the greater removal of uremic toxins by OL-HD has an impact in reducing of inflammation due to DC modulation. Another source of the non-antigen dependent pathway activation of DCs could be stress of patients on C-HD due to an increase of sympathetic nerve activity [12]. In this sense, renalase is a protein that oxidizes circulating catecholamines regulating heart rate and blood pressure which translate to sympathetic SNS activity. In this study, patients showed low renalase levels when they were submitted to C-HD and after four months of OL-HD their serum levels of renalase increased. These results corroborate that less inflammation leads to increased renalase levels, achieving values similar to those in healthy volunteers. This effect was robust, and probably the lower inflammatory profile could bring to an epinephrine via α-adrenoceptor/NF-κβ lower stimulation [11, 12]. Also, in this study, patients showed better control of Blood Pressure and less hypotensive events were noted, showing a better control of SNS [22, 33]. Those findings positively correlated with higher levels of renalase levels.

In the inflammatory field, several studies have demonstrated the immunosuppressive effects of vitamin D_3_ in the functions of DCs [41–48]. In this sense calcidiol levels showed a tendency to be higher in OL-HD patients compared to those on C-HD although values were not statistically different. It has been described that calcidiol or vitamin D suppresses the maturation of DCs.

Recently, the MPO (Membrane Permeability Outcome) study highlighted that survival was significantly increased in high-flux vs low-flux membranes in high-risk patients with serum albumin ≤4 g/dL and diabetic patients [36]. In this sense, the impact of OL-HD in diabetic and non-diabetic patients was evaluated. Dendritic cell maturation in diabetic C-HD was higher than in non-diabetic, this corroborates a basal inflammatory state in these patients. In fact, DC maturation in diabetic C-HD was corrected after the change of technique to OL-HD, achieving similar values to non-diabetic OL-HD, suggesting a correction in basal inflammation state in diabetic HD patients. These results are physiopathologically supported by the findings reported in MPO [36], ESHOL [33] and other studies [34]. Furthermore, these results were in concordance with T cell proliferation. Diabetic patients under C-HD showed higher percentage of lymphocyte proliferation on CFSE compared to diabetic patients under OL-HD. These differences were not so appreciated as much in non-diabetic patients.

In summary, the novelty of this study reveals functional improvement in inflammatory state in patients with a technique-switching to OL-HD, being more notable in diabetic patients. The originality of these results arises from an OL-HD-modulation of the immuno-inflammatory state mediated by DC maturation-down-regulation and control of the SNS improving renalase levels in HD patients.

## Supporting Information

S1 Table Base data collection from clinical and experimental parameters. (XLS)
